# Primary Idiopathic Osteoarthropathy: Could It Be Related to Alcoholism?

**DOI:** 10.1155/2017/2583762

**Published:** 2017-01-02

**Authors:** Yanal Alnimer, Suresh Subedi, Thair Dawood, Ghassan Bachuwa

**Affiliations:** Internal Medicine Department, Hurley Medical Center, Flint, MI, USA

## Abstract

*Background*. Hypertrophic osteoarthropathy (HOA) is a syndrome characterized by abnormal proliferation of the skin and bony structures at the distal extremities resulting in digital clubbing, periosteal bony reaction, and joint effusion. It can be primary (idiopathic) without any clear identifiable etiology as well as secondary to variety of systemic diseases most notably lung pathology.* Case Presentation*. We describe a rare case of primary idiopathic osteoarthropathy in a male patient who presented with severe pain and tenderness in his legs. His history was significant for long standing alcoholism. Physical examination showed severe fingers and toes clubbing. He reported similar changes in his mother. Clinical and radiological findings were remarkable for distal leg tenderness and diffuse periosteal bony reactions, respectively. Computerized tomography scan failed to show any pathology apart from fatty liver infiltration. In the absence of obesity or diabetes, this was consistent with alcoholic steatosis. He was started on nonsteroidal anti-inflammatory drug which dramatically improved his symptoms.* Conclusion*. Primary hypertrophic osteoarthropathy should be considered in a previously healthy person presenting with bony pain and finger clubbing especially after ruling out the common secondary causes. Moreover, alteration of prostaglandin metabolism secondary to alcoholic consumption might be a contributing factor.

## 1. Background

Hypertrophic osteoarthropathy (HOA) is a syndrome characterized by abnormal proliferation of the skin and bony structures at the distal extremities manifested by digital clubbing, periosteal bony reaction and joint effusion [1]. It is classified as either primary or secondary; the primary form is considered uncommon and accounts for about 3–5% of all cases. Overproduction of prostaglandin E2 (PGE2) is the proposed mechanism for the primary form. Mutations at hydroxy-prostaglandin dehydrogenase enzyme which is responsible for PGE2 degradation or at SLCO2A1 which is PGE2 transporter result in increase of serum levels of PGE2 that stimulate various intracellular signaling pathways including vascular endothelial growth factor expression (VEGF). The latter stimulates various cellular components of fibrous and bony tissues such as osteoblast, osteoclast, and fibroblast that lead to various bony and dermatological manifestations of hypertrophic osteoarthropathy [2].

Here, we describe a rare case of primary idiopathic osteoarthropathy in a male patient who presented with severe bilateral lower leg pain and tenderness. Clinical and radiological findings were significant for severe digital clubbing and periosteal bony reactions, respectively. Workup was negative for identifying a possible cause. Primary hypertrophic osteoarthropathy should be considered in a previously healthy person who presents with bony pain and finger clubbing.

## 2. Case Presentation

A 55-year-old male not known to have any previous medical problems presented with worsening bilateral lower leg pain which started couple days prior to his presentation for the first time in his life. He also reported chronic deformities in his fingers, toes, and nails which were present since his teenage, became worse as he grew up, and later stabilized. He denied any pain related to them until this presentation. He also denied any morning stiffness, hand joints pain, recent diarrhea, fever, or chills. He reported drinking alcohol since the age of 12, and he currently drinks 2-3 cans of beer per day. He had no history of smoking or illicit drug use. Family history was significant for similar hand/nail changes in his mother though he does not remember the age at which it started.

**Figure 1:**
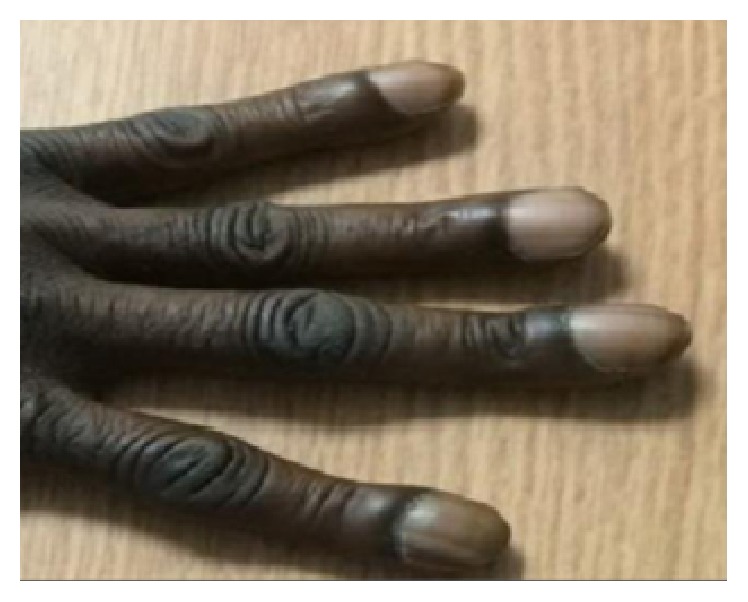
Severe digital clubbing.

**Figure 2:**
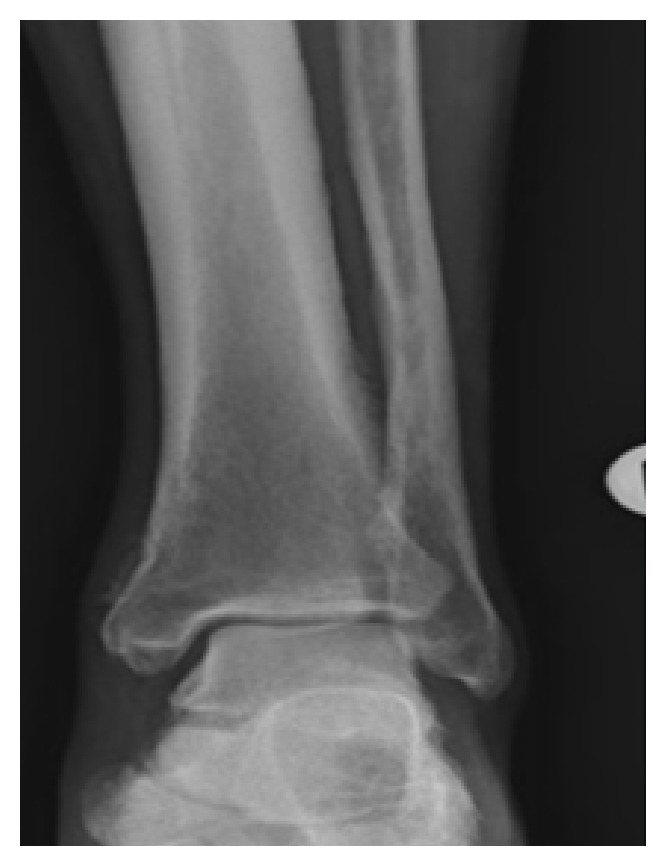
Course periosteal thickening along distal tibia and fibulae.

**Figure 3:**
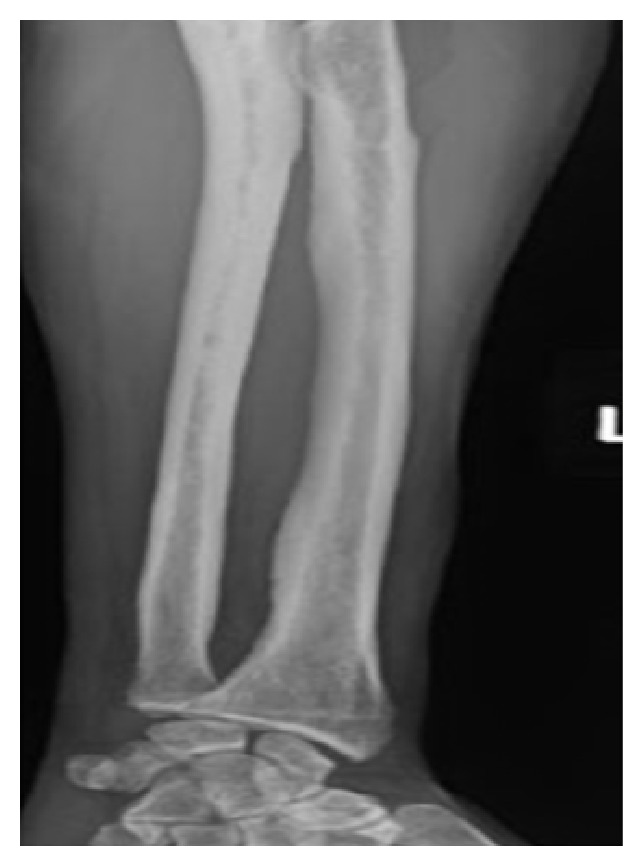
Marked cortical thickening of the radius and ulna.

Upon physical examination, the patient looked well-nourished; his vital signs were within normal limits. He was found to have severe bilateral clubbing of the fingernails and toenails along with osteoarthropathic changes in the hand joints (as illustrated in Figure 1). He had severe tenderness at the distal end of his legs, tenderness at the knee joints bilaterally, and mild left knee effusion. He had an antalgic gait secondary to the pain. Heart, lung, and abdominal examinations were unremarkable. There was no lymphadenopathy or hepatosplenomegaly. His skin examination was unremarkable. The patient's blood count and basic metabolic panels were within normal limits. Liver function tests were normal. An x-ray of his legs showed coarse periosteal thickening along the distal ends of the tibia and fibulae (as shown in Figure 2); knee and forearm x-rays (Figure 3) showed periosteal reaction along the long bones with left knee effusion. A chest x-ray and CT scan could not identify any lung pathology or masses. Diffuse fatty infiltration of the liver was noted without evidence of cirrhosis. Due to the presence of severe digital clubbing (Grade 4 as it resembles drumstick appearance) and diffuse long bone periostitis with no apparent secondary cause, the patient was diagnosed to have primary hypertrophic osteoarthropathy, which is also called pachydermoperiostosis. He responded well to NSAIDs and was discharged home in stable condition.

## 3. Discussion

Both primary and secondary forms have been identified as a cause for HOA. A variety of diseases, including cyanotic heart disease [3], pulmonary metastasis, and notably lung cancer [4], have been recognized as etiologies of the secondary form. Liver cirrhosis has been identified as a culprit in other cases as well [5].

The standard treatment of secondary HOA relies upon removing the underlying cause; however, this is not always curative. Ito et al. found only half of the cases of HOA secondary to lung cancer improved symptomatically and objectively with treatment [6].

The ability of the megakaryocyte to escape degradation in the pulmonary vascular bed due to an underlying lung pathology (lung cancer) or pulmonary arteriovenous shunt (as in the case of liver cirrhosis or cyanotic heart disease) results in platelet clumps at the capillary vascular bed with a subsequent release of a variety of cytokines, including fibroblast growth factor, platelet-derived growth factor, and vascular endothelial growth factors. This results in activation of the platelets and endothelial cells with subsequent proliferation of the skin and osseous structures [7].

Primary hypertrophic osteoarthropathy or pachydermoperiostosis (PDP) (other names include idiopathic hypertrophic osteoarthropathy or Touraine-Solente-Gole syndrome) is a rare disorder that shares some of the manifestations of the secondary form; however, it represents only 3–5% of all cases. Positive family history is found in 25–38% of the cases, which suggests PDP has familial and idiopathic forms. Castori et al. reviewed 204 patients in 68 published families with PDP and found 37 families had autosomal dominant inheritance while autosomal recessive inheritance was suggested in the rest. Autosomal dominant families showed a variable degree of expression and penetrance. No clear phenotypic differences have been found between the two modes of inheritance [8]; however, Latos-Bielenska et al. proposed that autosomal recessive cases tend to be more severe [9]. Sayli et al. found early onset skin ulcers, acrolysis of distal extremities, and growth retardation present in some of these cases. Nevertheless, the disease usually starts during adolescence and progresses insidiously over a period of 5–10 years before it stabilizes [10].

The familial form of PDP has been mapped to chromosome number 4 (4q33-q34), which encodes the hydroxy-prostaglandin dehydrogenase enzyme. Homozygous and combined heterozygous mutation of this enzyme increases the level of PGE2 through impairment of its degradation process; on the other hand, mutation at SLCO2A1 results in impairment of PGE2 cellular uptake. Both of these mutations result in high PGE2 levels which stimulate various intracellular signaling pathways including VEGF which eventually leads to various clinical manifestations of PDP [1, 2].

A wide spectrum of clinical manifestations has been described in the literature. The periosteal bony reaction that occurs at distal tubular bones, particularly the tibia and fibula (less frequently at the radius and ulna), which sometimes leads to adjacent joint effusion is one of the striking features. This periosteal reaction results in symmetrical irregular periosteal hypertrophy and new bone formation between the periosteum and the original cortex, which later fuse together. Distal ends of the tubular bones are most commonly affected, whereas the skull, vertebrae, and articular surfaces tend to be spared in most of the cases [11].

The dermatological manifestations are usually in the form of thickening of facial skin and dorsum of the hands and feet and range from mild to severe skin thickening and puckering. Finger clubbing is present in almost all cases. Pachyderma, hyperhidrosis, and seborrhea are frequently encountered skin changes, while folliculitis is less commonly encountered [12].

Microscopically, these changes result from fibroblast proliferation, thickening, and packing of collagen fibers resulting in acanthotic dermis as well as endothelial cell hyperplasia leading to partial occlusion of the blood vessel lumen. These skin changes are prominent in primary hypertrophic osteoarthropathy while bony changes tend to be more severe in the secondary form [12, 13].

Due to a wide variety of disease manifestations, different diagnostic criteria have been proposed. Touraine et al. recognized three forms of pachydermoperiostosis: a complete form characterized by both pachyderma (thickening of the skin) and periostitis, an incomplete form without pachyderma and a third form having pachyderma with minimal skin changes (Forme Fruste) [14]. However, Borochowitz suggested the diagnosis should be made only if two of the following have been identified: family history, digital clubbing, bony pain/radiographic changes, and hypertrophic changes of the skin [15].

Our patient presented with bilateral lower extremity pain and tenderness which started few days prior to his presentation for the first time in his life. The pain slowly got worse waking him up from sleep and affecting his gait. Radiological images showed diffuse periosteal bony reaction consistent with periostitis. Clinically, in addition to the tenderness, he had severe digital and toe clubbing which had a drumstick appearance (Grade 4) [16] which he said was present since he was a teenager. He did not have any facial features of HOA and he denied excessive sweating. Further questioning revealed his mother had similar finger clubbing changes and she had complained of joint pain too but he was not sure of the age of onset.

The patient was doing clinically well and his physical examination was completely normal apart from distal bony pain, tenderness, left knee effusion, and osteoarthropathic changes in his fingers and toes. His white blood cell count and metabolic panels were within normal limits. A CT scan did not identify any lung pathology or liver cirrhosis; however, it was remarkable for fatty liver infiltration. Of note, Farrús et al. and Mueller and Trevarthen have proposed that excessive alcohol intake with liver steatosis could trigger hypertrophic osteoarthropathy in the absence of significant liver damage or cirrhosis [17, 18]. On the other hand, excessive alcohol consumption alters prostaglandin metabolism and there is consensus data that PGE1 increases in alcoholic patients in the absence of liver cirrhosis [19]. Moreover, PGE1 has been suggested as a cause of hypertrophic osteoarthropathy [20].

The patient reports drinking alcohol since the age of 12; he has been drinking 2-3 cans of beer on a daily basis recently. CT scan showed extensive fatty liver infiltration. In the absence of diabetes and other features of metabolic syndrome, this was consistent with alcoholic steatosis. However, in the previous literature there was no strong association between alcoholic steatosis and osteoarthropathy as it is in liver cirrhosis. On the other hand, the increase production of PGE1 by excessive alcohol drink might be the trigger for our patient presentation.

The patient fits into the incomplete form of primary HOA category according to classification proposed by Touraine et al. He also meets 3 out of 4 of Borochowitz criteria. Nonsteroidal anti-inflammatory drugs are the cornerstone of treatment and intra-articular joint steroid injection, sulfasalazine, methotrexate, and tumor necrosis factor-alpha blockers can be used in resistant cases. The patient's symptoms responded dramatically to ibuprofen and he was discharged home after a couple of days [21].

## 4. Conclusion

Our case represents the incomplete form of primary hypertrophic osteoarthropathy, which accounts for 3–5% of all cases. It is vital to differentiate it from the secondary form, which requires treatment of the underlying cause. Moreover, the alteration of prostaglandin metabolism due to either excessive alcohol intake or fatty liver disease might trigger bony changes in susceptible patients. Nonsteroidal anti-inflammatory drugs remain the mainstay of treatment and other immunosuppressive agents can be used in resistant cases.
